# The effects of coronavirus disease 2019 pandemic on the South African health system: A call to maintain essential health services

**DOI:** 10.4102/phcfm.v12i1.2480

**Published:** 2020-07-22

**Authors:** Juliet Nyasulu, Himani Pandya

**Affiliations:** 1 School of Clinical Medicine, Division of Community Paediatrics, Faculty of Health Sciences, University of the Witwatersrand, Johannesburg, South Africa; 2 AFRIQUIP, Health Systems Strengthening, Johannesburg, South Africa

**Keywords:** COVID-19 pandemic, WHO health systems framework building blocks, health systems, essential services, HIV, EPI

## Abstract

South Africa had its first coronavirus disease 2019 (COVID-19) case on 06 March 2020 in an individual who travelled overseas. Since then, cases have constantly increased and the pandemic has taken a toll on the health system. This requires extra mobilisation of resources to curb the disease and overcome financial loses whilst providing social protection to the poor. Assessing the effects of COVID-19 on South African health system is critical to identify challenges and act timely to strike a balance between managing the emergency and maintaining essential health services. We applied the World Health Organization (WHO) health systems framework to assess the effects of COVID-19 on South African health system, and proposed solutions to address the gaps, with a focus on human immunodeficiency virus (HIV) and expanded programme on immunisation (EPI) programmes. The emergence of COVID-19 pandemic has direct impact on the health system, negatively affecting its functionality, as depletion of resources to curb the emergency is eminent. Diversion of health workforce, suspension of services, reduced health-seeking behaviour, unavailability of supplies, deterioration in data monitoring and funding crunches are some of the noted challenges. In such emergencies, the ability to deliver essential services is dependent on baseline capacity of health system. Our approach advocates for close collaboration between essential services and COVID-19 teams to identify priorities, restructure essential services to accommodate physical distancing, promote task shifting at primary level, optimise the use of mobile/web-based technologies for service delivery/training/monitoring and involve private sector and non-health departments to increase management capacity. Strategic responses thus planned can assist in mitigating the adverse effects of the pandemic whilst preventing morbidity and mortality from preventable diseases in the population.

## Introduction

In South Africa, since March 06, when the first coronavirus disease 2019 (COVID-19) case was reported, cases have increased to over 188 000 at the time of writing this article.^1^ This pandemic has called for extra mobilisation of resources to curb the disease and overcome financial loses whilst providing social protection to the poor.^2^ For decades, the South African health system has shouldered a quadruple burden of diseases (much of which is preventable), with mother and child health indicators far from accepatable targets.^3,4^ The South African primary healthcare provides both curative and preventive health services. These include under-five child health services, such as growth monitoring and expanded programme on immunisation (EPI); reproductive health services such as family planning, cervical and breast cancer screening, antenatal, labour and postnatal care services; chronic disease care for both communicable and non-communicable diseases including human immunodeficiency virus (HIV) services; and many other health promotion, preventative and curative services.^5^

It is concerning that out of 7.7 million HIV-positive people living in South Africa, about 4.9 million people are on antiretroviral treatment (ART), with one-third (1.6 million) not virally suppressed.^6^ The current evidence indicates that those virally suppressed are not at higher risk. However, in addition to 1.6 million, we do not know about the other 2 million not on ART, who probably will be at higher risk of severe COVID-19 infection.^6,7^ South Africa has existing antiretroviral (ARV) and vaccine stock-out challenges because of supply chain constraints.^8,9,10,11^ In addition, gaps have been identified around low routine immunisation coverage resulting in outbreaks of vaccine preventable diseases, such as measles, in South Africa.^12^ Anecdotal reports show that there is a decline in access to ART from March 2020 by those already initiated on ART. For example, some districts supported by Right to Care (RTC), a President’s Emergency Plan for AIDS Relief (PEPFAR) partner for Gauteng Province, show increasing numbers of missed appointments to collect ART.^13^ Similarly, EPI and other essential services are also likely to be affected. Fear of contracting COVID-19, the physical distancing policy and a shift in focus of service providers from basic essential services to COVID-19 pandemic demands may be the reasons for the decline in access to essential services.^14^

Emergence of the COVID-19 pandemic risks the worsening of existing gaps and increasing deaths as shown in the previous Ebola outbreaks.^15,16^ The resilience of health system to timely adapt and strike a balance between maintaining routine services and coping with the pandemic is crucial to mitigate the damage.^17^ Currently, there is a rapid and overwhelming increase in strain on the health system because of COVID-19, overstretching the capacity of healthcare workers to operate effectively.^18,19^ This article looks at the possible effects of COVID-19 pandemic on the South African health system and proposes possible solutions to maintain the delivery of essential health services whilst fighting the pandemic, with a specific focus on HIV and EPI. We believe that maternity care, child care (e.g. immunisation) and HIV services stand out as the most critical markers and a proxy for the strength of a health system, particularly in the context of South Africa.^6,8,12^ Resilience of a health system is indicated by its ability to offer basic healthcare services to pregnant women, children and people with HIV. These groups are comparatively more vulnerable and contribute to a high burden of morbidity and mortality at a population level. Lessons from previous epidemics such as Ebola have shown that when there is a threat to a health system, these groups are affected first and to a higher extent.^16^ Moreover, because of our experience and expertise in working with HIV and immunisation, we selected them as priority services and focus of this article.

## Approach

**FIGURE 1:**
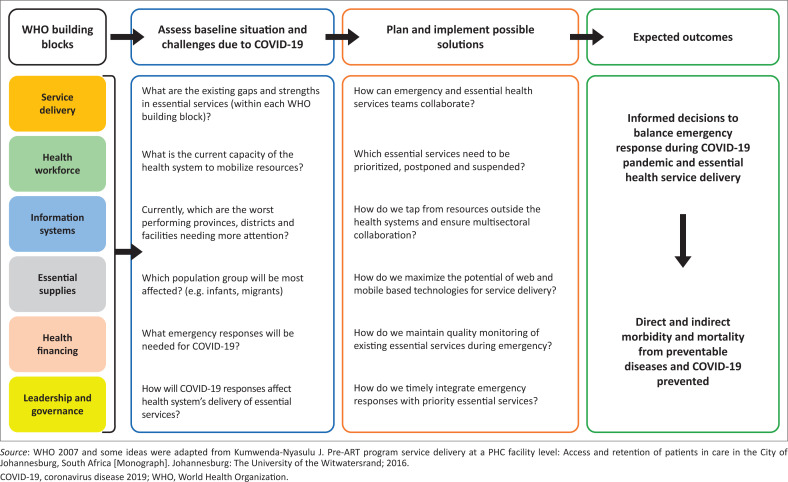
Conceptual framework (based on World Health Organization building blocks of a health system).

We applied the World Health Organisation (WHO) health systems framework and its six building blocks to assess how COVID-19 pandemic has affected the South African health system (Figure 1).^20^

Using the documented existing service delivery gaps, we analysed EPI and HIV programmes as examples of priority essential health services to be maintained by South Africa during this emergency period.^6,8,18^ In addition, solutions to strike a balance between responding to COVID-19 pandemic and maintenance of these essential services are proposed.

### Ethical consideration

This article followed all ethical standards for a research without direct contact with human or animal subjects.

## Results

We applied the WHO health systems framework to highlight strengths and gaps in the EPI and HIV service delivery system and explain how these are affected with the emergence of the COVID-19 pandemic. We have also proposed possible solutions on how to deal with these challenges. The questions raised in the above conceptual framework do not specifically direct our analysis; rather, they serve as a generic guideline for consideration by health managers and stakeholders in order to maintain primary health services during a pandemic. Table 1 summarises these gaps and possible solutions to maintain essential service delivery, with a focus on HIV and EPI.

**TABLE 1:** Health system risks posed by coronavirus disease 2019 and possible solutions to maintain essential services (according to the World Health Organization health systems framework).

Possible health system risks posed by COVID-19		Proposed solutions to maintain essential health services whilst responding to the pandemic
**1.**	**Service delivery (primary care level only)**	
	a. Essential services not prioritised because of competing interests,^18^ e.g., immunisation campaigns paused	World Health Organization proposes outreach mechanisms to ensure delivery of essential services,^18^ including immunisations and HIV services. For example, auxiliary nurses can do field-based immunisations (at rural health posts closer to community) rather than children crowding at clinics. Human immunodeficiency virus experts advise that male circumcision can be paused whilst harm reduction and condom distribution and HIV treatment services need to be maintained with modifications that will reduce contact with service providers.^21^ Currently, South Africa is implementing Central Chronic Medicines Dispensing and Distribution (CCMDD) programme whereby stable patients collect their chronic medications at different pick-up points near them outside the health facility. We recommend that CCMDD must be maximised to reduce physical contacts with service providers.^22^ Immunisation campaigns can be modified to reduce huge numbers at once. Consider integrated community-based outreach platforms offering immunisation services.^23^
	b. Covid-19 Physical distancing policy compels population to defer healthcare seeking for essential routine services like HIV and EPI^14^	Integrate essential services with COVID-19 services at facility and community levels. For example, involve nurses delivering EPI and HIV services in screening for COVID-19 and reporting cases.^23^ Identify and prioritise vulnerable communities including infants, poor and the elderly for essential services. For example, maximise the use of social protection grants available during the emergency to promote access by the vulnerable groups. Generate a country-specific list of essential services for SA (based on context and supported by WHO guidance and tools). Prioritise current worse-performing provinces, districts and facilities, which need more attention and resources for delivery of essential services. Shift focus from conducting face-to-face, manual and paper-based routine operations and monitoring to utilising information technology and web-based platforms for maintaining services, for example, health promotion and prevention messages through mobile technology. Ensure positive health-seeking behaviour and adherence to care by maintaining population’s trust in the capacity of the health system, to safely meet essential needs and to control infection risk in health facilities. The communities should be sensitised and reassured through media, text messages and platforms like religious and other existing community structures. (WHO operational guidelines)^18^
**2.**	**Human resources**	
	a. Health workers infected with COVID-19	Intensive COVID-19 screening for health service providers. Prioritise and ensure adequate supply of personal protective equipment (PPE) for health workers.
		Explore ways to support those needing self-isolation and quarantine whilst protecting their family/household.
	b. Lack of COVID 19 training for health workers	Consider short, web-based training for health workers in COVID-19 screening, first-line treatment, referral guidelines, quarantine/isolation policies and personal protection through smart phones (based on videos/apps). They also need to be trained on how to assure/motivate/counsel the clients because they are the frontline contacts.
	c. Shortage of staff from essential services because of redeployment towards COVID-19 response	Consider task shifting and scope expansion where possible to improve access to care (24) – for example, enrolled nurses and enrolled assistant nurses could take up health prevention/promotion as well as curative tasks from professional nurses, for example, immunisation. Use of qualified health workforce resident in South Africa but not working, to be recruited. Part time health workforce to be asked to work full time. Utilise the senior health workforce students from training institutions to alleviate staff shortage pressures. Maximise health workforce from the non-governmental partners like provincial and district PEPFAR collaborations, defence, Red Cross, etc. Clinical associates, senior students from nursing colleges and interns can be deployed on a short-term basis and, if possible, accelerate early certification without compromising quality. Redistribute and redeploy staff from non-affected areas, or high-performing districts to low-performing districts.
	d. Health workforce overwhelmed, at risk of resignations	Reassurance from department of health, small incentives for those health workers who contribute to both PHC and COVID-19 response. Explore ways of acknowledging and appreciating the health workforce.
**3.**	**Health information systems**	
	a Worsening of the quality of existing data in public health system^25^	Minimise paper-based reporting and data collection considering physical distancing Strengthen online, web-based information systems for monitoring and progress of HIV and EPI programmes, which can be directly used by health workers and data can be submitted through smart phones to a centralised server, which is accessible to all project managers and decision-makers.
	b. Competing interests leading to a shift in focus to monitor the COVID-19 data currently in greatest demand	During the emergency, ensure monitoring of ongoing delivery of essential health services to identify gaps and provide timely response. Prioritise, in this case, EPI, HIV and other critical indicators in the DHIS that need to be essentially monitored and leave out those indicators the monitoring of which can be delayed, such as male circumcision.
	c. Lack of time for quarterly reviews to monitor progress on essential services to identify and address gaps, for example, health facility assessments, IMCI health worker supervision, etc.	Decentralise quarterly reviews at facility level – promote internal reviews of routine essential services (designate a team of nurses led by facility managers) if supervisors cannot visit the clinics and provide online feedback to managers. Web-based data reviews through Zoom/MS team/Skype/Google Meet, etc., and other platforms will save time without disturbing physical distancing.
	d. Surveillance and reporting of AFP and vaccine preventable diseases might not be ensured	Maximise online tools for monitoring and reporting of cases of acute flaccid paralysis (AFP) for polio, measles, etc. (e.g. apps, web-based software) Involve private clinics and GPs in reporting and surveillance.
**4.**	**Access to essential medicines**	
	a. South Africa has existing ARV and vaccine stock-out challenges because of supply chain constraints.^8,9,10,11^	Prioritise the worst-performing provinces on ARVs, vaccines and other essential medicines stock-outs. Collaborate with private health sector, pharmaceutical companies to maximise contribution and utilise their platforms. Use of advances in technology to improve supply chain management could be linked with current initiatives such as MomConnect. Stock-outs for medicines and vaccines can be reported by facilities or districts online through web-based platforms which are monitored by the district supply chain managers and supplies could be procured accordingly. For example, Blood Information and Management Application (BIMA) in Bangladesh takes online demand for blood and manages procurement.^26^
	b. Shortage of COVID-19 essential protective wear for healthcare workers has already been reported^27^ as manufacturers fail to meet demands	Enhance and promote local manufacturing of PPEs. Capitalise on buffer system.
**5.**	**Health financing**	
	Economy shrinking coupled with high financial constraints to cope with the pandemic may lead to fiscal constraints on essential health services spending for HIV and EPI^28^	Presidency and department of finance need to coordinate with department of health and decide on diverting any funds available in contingency or from other non-essential departments, for example, tourism, and create extra budget heads for maintaining essential health services such as procuring ARVs or vaccines. Divert surplus funds under HIV and EPI heads towards poor performing districts and provinces for extra support (e.g. run a mobile unit for vaccination or conduct a community-based catch-up campaign). Strengthen private and public health sector partnership to ensure that the public health system taps from the available resources in the private sector. Initiate and promote COVID-19 fundraising activities at local, regional and national levels. For example, SA has introduced solidarity fund where individuals and firms are donating resources to meet the needs of the poor, and at the time of writing this article, R2.5 billion had been raised with a target of R4bn.^29^
**6.**	**Leadership and governance**	
	a. Depleted leadership capacity for essential services as programme managers had been redeployed to COVID-19.	Inter-sectoral collaboration – human resources from other non-health departments need to be involved to provide the required leadership and coordinate with health department. These could include Department of Finance, Department of Agriculture, Department of Education, NGO and multi-national partner institutions, for example, UNICEF and WHO. The use of other ministerial departments to complement the containment of the pandemic. For example, the Ministry of Water and Sanitation to ensure that population including the hardest to reach ones have access to clean water and soap for handwashing. This can be accomplished in collaboration with the Ministry of Defence that can help in distribution. In addition, the Ministry of Information and Education can support free online education, the Ministry of Telecommunications can generate awareness by media campaigns and Telkom companies can be involved to provide mobile data free of cost to support information exchange and online management of health information, etc. Department of Health can also utilize senior students from medical, nursing and public health universities, clinical associates, interns and paediatrics registrars to assist with programme management and operations. They are better trained and equipped and can work in coordination with existing programme managers and leaders on a short-term voluntary basis to get a hands-on experience in public health and emergency response.
	b. Decisions to navigate and strike a balance between the emergency COVID-19 and essential services	Close collaboration between the COVID-19 and essential services teams at all levels of management (national, provincial, district, sub-district and below) to identify and agree on the priority essential services that must maintain continuity during emergency period. National coordinators for HIV and EPI need to adapt and implement WHO essential services guidelines to South African context and communicate with provincial and district-level programme managers on how to operationalise the modified guidelines in their respective areas.

*Source:* WHO 2007 and some ideas were adapted from Kumwenda-Nyasulu J. Pre-ART program service delivery at a PHC facility level: Access and retention of patients in care in the City of Johannesburg, South Africa [Monograph]. Johannesburg: The University of the Witwatersrand; 2016.

CCMDD, Central Chronic Medicines Dispensing and Distribution; COVID-19, coronavirus disease 2019; EPI, expanded programme on immunisation; PPE, personal protective equipment; BIMA, blood information and management application; AFP, acute flaccid paralysis; SA, South Africa; WHO, World Health Organization; PEPFAR; DHIS; PHC; IMCI; GPs; ARV; PPEs; UNICEF; NGO.

## Discussion

The emergence of the COVID-19 pandemic has put great burden on the health system, negatively affecting its functionality. We propose the WHO health systems framework as an approach for assessing and prioritising services by health systems to strike a balance between the responses to COVID-19 pandemic and delivery of quality essential healthcare services, with a focus on EPI and HIV programmes.

Firstly, representation and close collaboration between the COVID-19 and the essential services teams at all levels are recommended. These teams together will need to identify priority essential services within the two programmes and decide which services are to be continued, postponed or suspended.^18^ At this time, identification of how the emergency is affecting the health system and which geographic areas and vulnerable groups should be prioritised are critical.^18,30^ Application of the WHO health systems building blocks will provide a systematic and comprehensive approach to the identification of these gaps.^20^

The next step is to identify and implement solutions to address the gaps worsened or caused by the COVID-19 emergency response. For instance, the redeployment of health workforce, coupled with others being infected with COVID-19, depleted the already existing shortage.^19^ Therefore, considering task shifting, integration of services, utilisation of senior students, tapping from NGO partners and government workforce outside the Department of Health would alleviate the health workforce shortage.^18,19,20,21,22,23,24^ The most important task is to provide support to the available health workforce in different aspects needed.^31^

Admittedly, the COVID-19 physical distancing currently advocated for puts HIV-positive individuals and parents in a dilemma to defer routine appointments, worsening the current gaps in HIV/EPI programme.^21^ Therefore, in order to ensure positive health-seeking behaviour and adherence to care, there is a need to maintain population’s trust in the capacity of the health system to safely meet essential needs and to control infection risk in health facilities.^18^ This requires training of health service providers in providing essential services with extra safety to control the spread of COVID-19,^18^ accompanied by communication with the users and reassurance of access and safety of health services. In times of such pandemics, it is important to ensure that the vulnerable communities including the poor and the elderly have the ability to access these essential services.^18,30^

Furthermore, existing gaps in immunisation and HIV services including stock-outs have been established, which can worsen when dealing with the COVID-19 pandemic.^8,9^ We therefore propose service delivery approaches that do not attract crowds like CCMDD and outreach services.^18,22,23^ We also suggest the need to minimise relying on manual operations and paper-based service delivery and monitoring, and utilise information technology and web-based platforms to monitor and conduct its routine operations.^26,32^ End-user monitoring of the supply chain by patients and civil society has the potential to increase transparency and complement public sector monitoring systems.^8^

In conclusion, the resilience of the health system is a critical determinant of how a country responds to a pandemic.^33^ In emergencies like COVID-19, the ability of a health system to deliver essential services is dependent on the existing burden and baseline capacity of the health system. The existing high disease burden would put the South African health system in a fragile state to cope with the pandemic if timely adaptation actions are not taken. The approach proposed in this article, about using WHO building blocks to identify existing gaps, challenges and possible solutions, can be adopted by other low- and middle-income settings to identify priority actions in order to strike a balance between attending to a pandemic and simultaneously maintaining essential services. The authors envisage that applying these principles during such pandemics will lead to informed health systems decisions in striking a balance between emergency response and essential health service delivery, and maintaining of curative and preventive essential health services, which in turn will reduce morbidity and mortality from preventable and treatable diseases.
